# Estimated glucose disposal rate is associated with brain aging and dementia among diabetes-free older adults

**DOI:** 10.1093/gerona/glaf243

**Published:** 2025-10-31

**Authors:** Jiao Wang, Shuqi Wang, Abigail Dove, Sakura Sakakibara, Stéphanie Paillard-Borg, Marc Guitart-Masip, Jirong Yue, Weili Xu

**Affiliations:** National Clinical Research Center for Geriatrics, West China Hospital, Sichuan University, Chengdu, Sichuan, China; Department of General Medicine, Xinqiao Hospital, Chongqing, China; Aging Research Center, Department of Neurobiology, Care Sciences and Society, Karolinska Institutet, Stockholm, Sweden; Aging Research Center, Department of Neurobiology, Care Sciences and Society, Karolinska Institutet, Stockholm, Sweden; Department of Health Sciences, The Swedish Red Cross University, Huddinge, Sweden; Center for Psychiatry Research, Region Stockholm, Stockholm, Sweden; Center for Cognitive and Computational Neuropsychiatry (CCNP), Karolinska Institutet, Stockholm, Sweden; National Clinical Research Center for Geriatrics, West China Hospital, Sichuan University, Chengdu, Sichuan, China; Aging Research Center, Department of Neurobiology, Care Sciences and Society, Karolinska Institutet, Stockholm, Sweden

**Keywords:** Insulin resistance, Estimated glucose disposal rate, Dementia, Brain age, *APOE* ɛ4

## Abstract

**Background:**

To investigate the association of estimated glucose disposal rate (eGDR), a validated measure of insulin resistance (IR), with brain aging and dementia among diabetes-free people.

**Methods:**

This study included 258 732 diabetes- and dementia-free adults aged ≥55 from UK Biobank, including 15 389 participants who underwent brain MRI scans. eGDR was assessed by a well-established formula. Brain age gap (BAG) was calculated as difference between machine learning-predicted brain age and chronological age. Dementia was ascertained based on medical records. Data were analyzed using Cox, Laplace, and linear regression.

**Results:**

Over the follow-up, 7063 participants developed dementia. The hazard ratios of dementia for eGDR quartiles 2-4 compared to eGDR quartile 1 were 0.88 (0.81, 0.96), 0.83 (0.76, 0.92), and 0.73 (0.66, 0.82), respectively. High eGDR was further associated with 1.31 (0.81, 1.80) years later onset of dementia. Those with high eGDR had 2.09 (1.74, 2.45) years younger brain age than chronological age. Among *APOE* ɛ4 carriers, those with high eGDR had 14% lower incidence of dementia and a 1.77-year gap between brain age and chronological age (*p*_-for-interaction_ < .001).

**Conclusion:**

Higher eGDR is associated with prolonged onset of dementia and delayed brain aging among diabetes-free individuals, and could buffer genetic risk of *APOE* ɛ4.

## Introduction

Dementia is the fifth leading cause of death globally, with its prevalence continuing to rise.^1^ As there is currently no curative treatment for dementia, the management of modifiable risk factors is crucial to slow the progression of cognitive decline and delay the onset of dementia.^2^ Diabetes is associated with cognitive decline, dementia, and neurodegenerative brain pathology.^3^^,^^4^

Insulin resistance (IR) might be an important mechanism linking diabetes and dementia.^5^ Previous studies have reported that IR is associated with an increased risk of dementia in individuals with diabetes.^6^^,^^7^ In addition, growing evidence suggests that the increase in IR starts several years before the onset of diabetes or even prediabetes^8^ and that such an increase is associated with subsequent diabetes development.^8^^,^^9^ Pre-diabetes, an intermediate status between normal glucose regulation and diabetes with impaired glucose tolerance or fasting glucose,^8^ has also been linked to an increased risk of dementia^10^^,^^11^ and brain aging.^12^ However, whether IR also serves as an underlying mechanism for the association of pre-diabetes with dementia and brain aging remains unclear.

To date, only one study has reported the association between IR (assessed by McAuley index) and increased risk of dementia in a diabetes-free population,^13^ without considering pre-diabetes. Limited studies found that IR was associated with poor magnetic resonance imaging (MRI) markers of brain aging among the diabetes-free population, with inconsistent findings.^14–16^ The estimated glucose disposal rate (eGDR) is a well-established and validated surrogate of IR.^17^^,^^18^ The eGDR has been validated as having a good correlation with IR measured by direct insulin resistance methods—the hyperinsulinemic euglycemic glucose clamp,^19^ and in contrast, eGDR is more accessible and better suited for large-scale population studies.^18^ Compared to other epidemiological indices of insulin resistance, such as the triglyceride-glucose (TyG) index and its derivatives, HOMA-IR, HOMA-β, TG/HDL, QUICKI, and METS-IR, eGDR has shown stronger prediction for clinical outcomes, including cardiovascular disease, metabolic syndrome, etc.^20^^,^^21^ Furthermore, eGDR provides an accurate assessment of IR in diabetes-free individuals.^22^ However, the association of eGDR with incident dementia and brain aging has not been thoroughly evaluated.

To fill this knowledge gap, in the present study of 16-year longitudinal data from more than 250 000 older adults in the UK Biobank, including more than 15 000 who underwent brain MRI, we aimed to examine the association of eGDR with dementia risk and brain aging in older adults, considering the glycemic status including normoglycemia and pre-diabetes using data from a large community-based longitudinal study of the UK Biobank.

## Methods

### Study design, setting, and participants

The UK Biobank is a large-scale community-based longitudinal study that enrolled 502 353 UK residents aged 37-73 years at baseline.^23^ Between 2006 and 2010, participants underwent a baseline examination consisting of physical and clinical assessments and a series of touchscreen-based questionnaires to collect information on sociodemographic and lifestyle factors. Approximately 9 years later, between 2014 and 2020, over 40 000 participants underwent a brain MRI scan.

Selection of the study population is illustrated in Supplementary Figure S1. The sample was initially restricted to 466 317 participants who were free from diabetes (type 1 or type 2) at baseline. We further excluded participants with prevalent dementia (*n* = 573), baseline age <55 years (*n* = 94 725), or missing information on eGDR-related factors (*n* = 36 423), leaving a sample of 258 732 participants for the main analyses (Supplementary Figure S1). The neuroimaging subsample consisted of 15 389 participants who underwent a brain MRI scan over the follow-up period and were free from chronic brain disorders at the time of MRI assessment (Supplementary Table S1). All data used in this study were obtained from the UK Biobank (http://www.ukbiobank.ac.uk) through application 67048.

### Exposure

eGDR was calculated according to the following validated formula^17^^,^^18^: eGDR (mg/kg/min) = 21.158 − (0.09 × waist circumference [cm]) − (3.407 × hypertension [yes = 1/no = 0]) − (0.551 × HbA1c [%]). Higher values of eGDR are more favorable and represent a faster rate of insulin processing (ie, lower IR). eGDR was considered as both a continuous and categorical variable (grouped into quartiles; quartiles 1-4).

### Outcomes

In the present study, the onset of dementia and BAG were used as the main outcomes. The analysis of BAG excluded people with any chronic brain disorders (including dementia), which serve as a phenotype for pre-clinical brain pathological changes in dementia.

#### Assessment of dementia

Prevalent and incident dementia (including Alzheimer’s disease [AD] and vascular dementia [VaD]) was ascertained based on information from self-report, medical records (Supplementary Table S2), and death records from the Hospital Episode Statistics (England), the Scottish Morbidity Record (Scotland), and the Patient Episode Database (Wales).^24^ Primary and secondary hospital diagnoses and causes of death were recorded using the International Classification of Diseases (ICD-9 and 10) coding system. The ICD codes used to identify dementia were selected and validated by the UK Biobank outcome adjudication group. Follow-up time was calculated as the time (in years) from study entry to dementia diagnosis, death, or the end of follow-up (October 10, 2023), whichever occurred first.

#### Assessment of brain age

Brain age was modeled based on multi-modal neuroimaging phenotypes, as has been previously described in detail in our earlier publications.^12^^,^^25^ In brief, a total of 1079 individual phenotypes were generated through six different MRI modalities. A total of 9 different machine learning models were trained and tested to predict brain age; these included Least Absolute Shrinkage and Selection Operator (LASSO) regression, eXtreme Gradient Boosting (XGBoost), and Support Vector Regression (SVR) combined with three possible feature selection strategies (no feature selector, FeatureWiz, and RFECV).^26^ Ultimately, LASSO without feature selection was identified as the brain age prediction model with the minimum mean absolute error. Brain age gap (BAG) was defined as predicted brain age minus chronological age. A positive BAG indicates that the predicted brain age is older than chronological age, ie, accelerated brain aging; a negative BAG means that the predicted brain age is younger, that is, delayed brain aging.^27^^,^^28^

### Assessment of covariates

Socioeconomic status (SES) was measured using the Townsend Deprivation Index (TDI), an indicator of socioeconomic deprivation based on neighborhood levels of unemployment, household overcrowding, car non-ownership, and home non-ownership.^29^ Education level was self-reported and dichotomized as college or noncollege. Body mass index (BMI) was calculated as weight (kg)/height (m^2^) and was further categorized as underweight (<18.5 kg/m^2^), normal weight (18.5-24.9 kg/m^2^), overweight (25.0-29.9 kg/m^2^), or obese (≥30 kg/m^2^). Smoking and drinking status were categorized as never, former, or current smoker/drinker. Regular physical activity was defined as at least 150 min of moderate-intensity activity per week, 75 min of vigorous activity per week, or a combination of equivalent activities.^30^ Social contact was estimated based on responses to the question “How often do you visit friends or family or have them visit you?” These responses were further dichotomized as high (almost daily, 2-4 times a week, about once a week) or low level (about once a month, once every few months, never or almost never, or no friends/family outside household). Dyslipidemia was defined as high total cholesterol (>6.2 mmol/L), high LDL-C (>4.1 mmol/L), or high triglycerides (>2.3 mmol/L).^31^ Hypertension was defined based on self-report, blood pressure measurement (systolic ≥ 140 mm Hg, diastolic ≥ 90 mm Hg), or antihypertensive medication use. Cardiovascular disease (including myocardial infarction, angina, congestive heart failure, atrial fibrillation, and stroke) was ascertained based on self-report and medical records. Diabetes was defined as a medical record of diabetes, self-reported history of diabetes, glycosylated hemoglobin A1c (HbA1c) ≥ 6.5%, or the use of glucose-lowering medications.^32^ Among diabetes-free participants, pre-diabetes was defined as HbA1c 5.7% to 6.4%, and normoglycemia was defined as HbA1c <5.7%.^32^ Finally, the *apolipoprotein E* (*APOE*) gene (rs429358 and rs7412) was genotyped and dichotomized as carriers versus noncarriers of the ε4 allele.

### Statistical analysis

Baseline characteristics of the study population by eGDR quartiles were analyzed using chi-square tests for categorical variables and one-way ANOVA for continuous variables.

We performed two procedures to estimate the association between eGDR and incident dementia (including AD and VaD) among all participants, as well as participants with normoglycemia and pre-diabetes. First, a time-to-event analysis of Cox proportional hazards regression was used to estimate the hazard ratios (HRs) and 95% confidence intervals (CIs) for the incidence of dementia associated with eGDR. The proportional hazards assumption was tested using Schoenfeld residuals regressed against follow-up time; no violations were observed. In addition, models fitted with 4-knots at fixed percentiles of the eGDR distribution (25th [eGDR: 6.17], 50th [eGDR: 7.43], and 75th [eGDR: 9.98]) restricted cubic splines (RCS) were conducted to test for linear and nonlinear relationships between eGDR and dementia risk. Second, a flexible percentile regression—Laplace regression—was used to estimate percentile differences (PDs) and 95% CIs in years of onset for incident dementia. Given that only a small fraction—less than 10%—of participants developed dementia, we estimated differences in the median time (in years) until the first 10% of participants developed dementia. Linear regressions were used to estimate the β-coefficients and 95% CIs for the association between eGDR and BAG. Models were first basic-adjusted for age, sex, and education. Multivariable-adjusted models were further adjusted for socioeconomic status, BMI, smoking status, alcohol drinking, physical activity, social contact, dyslipidemia, cardiovascular disease, and *APOE* ε4. The least squares (LS) means and standard errors derived from the above multivariate adjustment models were used to represent the BAG for individuals with different eGDR levels.

Stratified analyses were performed to explore the role of sex (male vs female), physical activity (regular vs irregular), BMI (normal vs overweight vs obese), and *APOE* ε4 (carriers vs noncarriers) in the association between eGDR and dementia/BAG. Also, we performed joint exposure analysis by incorporating an eight-category indicator variable that combined eGDR (quartile 1-4) and *APOE* ε4 (carriers vs noncarriers) into the models to detect whether high eGDR could attenuate dementia-related genetic risk. Multiplicative interactions between eGDR and sex, physical activity, BMI, and *APOE* ε4 were assessed by incorporating the cross-product term into the models.

All statistical analyses were performed using Stata SE 16.0 (StataCorp LLC, College Station, TX). *p*-values < .05 were considered statistically significant.

#### Sensitivity analysis

We repeated the eGDR-dementia association analyses after: (1) performing a competing risk model with death as the competing event; (2) excluding participants with possible prodromal/undiagnosed dementia (ie, incident dementia cases that occurred during the first 3 years of the follow-up) to reduce potential reverse causality; and (3) performing multiple imputation for missing values of covariates using chained equations.

## Results

### Baseline characteristics of the study population

**Figure 1.**
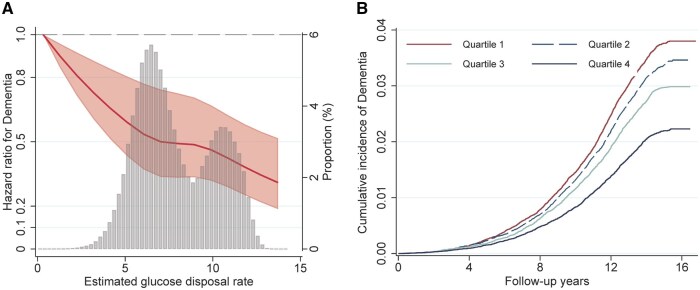
The association between estimated glucose disposal rate (eGDR) and dementia. (A) The nonlinear association between eGDR and the risk of dementia was tested by Cox regression with a restricted cubic spline. The association was linear (*p* for nonlinear > .05) after adjusting for age, sex, education, socioeconomic status, body mass index, smoking status, alcohol drinking, physical activity, social activity, dyslipidemia, cardiovascular disease, and apolipoprotein E ε4. Gray bars represent the distribution of eGDR in the study population. (B) The cumulative incidence of dementia by quartile of eGDR.

Baseline characteristics of the study participants are provided in Table 1 (mean baseline age 61.98 ± 4.07; 54.83% female). The distribution of eGDR ranged from −1.12 to 16.56 mg/kg/min, with a mean of 7.93 ± 2.24 mg/kg/min (Figure 1A). Compared to participants with eGDR quartile 1, those with higher eGDR were generally younger, female, white, and with higher education levels and socioeconomic status. They also exhibited lower BMI, engaged in regular physical activity, and were more likely to be nondrinkers, nonsmokers, and *APOE* ɛ4 carriers. In addition, they tended to have lower rates of dyslipidemia, cardiovascular disease, and pre-diabetes (Table 1). Characteristics of the neuroimaging subsamples are shown in Supplementary Table S3.

**Table 1.** Baseline characteristics of the study populations by estimated glucose disposal rate (eGDR) (*N* = 258 732).

Characteristics	eGDR				*p* Value
	Quartile 1 (*n* = 64 683)	Quartile 2 (*n* = 64 690)	Quartile 3 (*n* = 64 677)	Quartile 4 (*n* = 64 682)	
**Follow-up time (year)**	14.38 (13.53, 15.14)	14.49 (13.66, 15.20)	14.52 (13.69, 15.25)	14.57 (13.82, 15.31)	−
**eGDR (mg/kg/min)**	5.31 ± 0.73	6.77 ± 0.36	8.62 ± 0.79	11.03 ± 0.66	<.001
**Age (year)**	62.50 ± 4.05	62.56 ± 4.01	61.86 ± 4.06	61.00 ± 3.97	<.001
**Female**	19 105 (29.54)	33 389 (51.61)	40 347 (62.38)	49 029 (75.80)	<.001
**Townsend Deprivation Index**	−2.20 (−3.64, 0.44)	−2.45 (−3.78, −0.15)	−2.46 (−3.81, −0.16)	−2.48 (−3.83, −0.23)	<.001
**Education (college)**	15 670 (24.57)	17 688 (27.69)	19 415 (30.36)	23 019 (35.96)	<.001
**BMI (kg/m^2^)**	31.31 ± 4.26	26.87 ± 2.94	26.31 ± 4.04	24.34 ± 2.81	<.001
** Underweight (<20)**	1 (0.00)	105 (0.16)	2019 (3.13)	3337 (5.16)	<.001
** Normal (20-25)**	1282 (1.99)	16 693 (25.85)	24 766 (38.37)	36 332 (56.23)	
** Overweight (25-30)**	27 060 (41.95)	39 702 (61.49)	26 659 (41.30)	22 983 (35.57)	
** Obese (≥30)**	36 159 (56.06)	8064 (12.49)	11 106 (17.21)	1959 (3.03)	
**Alcohol drinking**					<.001
** Never**	2418 (3.75)	2556 (3.96)	2747 (4.25)	2762 (4.28)	
** Former drinker**	2444 (3.79)	2008 (3.11)	2290 (3.55)	2227 (3.45)	
** Current drinker**	59 672 (92.47)	60 009 (92.93)	59 537 (92.20)	59 565 (92.27)	
**Smoking status**					<.001
** Never**	28 021 (43.60)	34 164 (53.09)	34 757 (53.99)	37 040 (57.49)	
** Former smoker**	30 479 (47.42)	25 110 (39.02)	23 810 (36.99)	21 799 (33.84)	
** Current smoker**	5772 (8.98)	5074 (7.89)	5804 (9.02)	5586 (8.67)	
**Regular physical activity**	38 280 (66.23)	43 034 (73.60)	42 728 (72.39)	44 913 (74.88)	<.001
**High level of social contact**	34 473 (53.68)	33 572 (52.24)	34 096 (53.05)	33 989 (52.86)	<.001
**Dyslipidemia**	31 302 (50.81)	30 227 (48.90)	30 978 (50.16)	28 843 (46.63)	<.001
** TC (mmol/L)**	5.57 ± 1.19	5.85 ± 1.19	5.97 ± 1.10	6.01 ± 1.06	<.001
** LDL-C (mmol/L)**	3.52 ± 0.90	3.67 ± 0.91	3.73 ± 0.84	3.73 ± 0.81	<.001
** TG (mmol/L)**	2.12 ± 1.09	1.78 ± 0.91	1.68 ± 0.91	1.45 ± 0.75	<.001
**Cardiovascular disease**	9441 (14.60)	6027 (9.32)	3829 (5.92)	2000 (3.09)	<.001
**Pre-diabetes**	24 455 (37.81)	15 613 (24.14)	15 046 (53.26)	11 672 (18.05)	<.001
***APOE* ɛ4 carriers**	14 949 (28.05)	15 363 (28.36)	15 636 (28.68)	15 823 (28.72)	.049

Abbreviations: BMI = body mass index; TC = Total cholesterol; LDL-C = low-density lipoprotein cholesterol; TG = total triglyceride; *APOE* = *apolipoprotein* E.

Missing data: Education = 3107; TDI =235; Smoking = 1316; Alcohol consumption = 497; BMI = 505; Regular physical activity = 23 466; Regular social contact = 1689; Dyslipidemia = 11 690; *APOE* ɛ4 = 41 644.

*p* Values were tested using chi-square tests for categorical variables and one-way ANOVA for continuous variables.

### Association between eGDR and incident dementia

Over a median follow-up of 14.5 years (interquartile range: 13.6 to 15.2 years; 3 623 195.9 person-years), 7063 incident cases of dementia were identified (20.0 [95% CI: 19.5, 20.5] per 10 000 person-years, age- and sex- specified), including 3285 (9.3 [8.9, 9.6] per 10 000 person-years) with AD and 1428 (4.0 [3.8, 4.2] per 10 000 person-years) with VaD. The association between eGDR and the risk of dementia was linear (*P*_nonlinear_ >0.05, Figure 1A). This was visually supported by Kaplan–Meier curves showing progressively lower cumulative dementia incidence with increasing eGDR quartiles (Figure 1B).

Higher eGDR was dose-dependently related to lower risk of all-cause dementia (HR: 0.95 [0.93, 0.97]) (Table 2). When analyzed by quartile, the HRs for dementia were 0.88 (0.81-0.96) for quartile 2, 0.83 (0.76-0.92) for quartile 3, and 0.73 (0.66-0.82) for quartile 4, compared to quartile 1. Similar patterns were observed for AD and VaD (Table 2).

**Table 2.** Hazard ratios (HRs) and 10th percentile differences (PDs) in years of onset for incident dementia in relation to estimated glucose disposal rate (eGDR).

eGDR	No. of participants	**Dementia** (cases = 7063)		Dementia subtypes			
				**Alzheimer’s disease **(cases = 3285)		**Vascular dementia **(cases = 1428)	
		HR (95% CI)^a^	10th PD (95% CI)^a^	HR (95% CI)^a^	10th PD (95% CI)^a^	HR (95% CI)^a^	10th PD (95% CI)^a^
**All participants**							
**Continuous**	258 732	**0.95 (0.93, 0.97)^b^**	**0.22 (0.15, 0.30)^b^**	**0.96 (0.94, 0.99)^b^**	**0.17 (0.06, 0.27)^b^**	**0.90 (0.87, 0.94)^b^**	**0.42 (0.25, 0.59)^b^**
**Categorical**							
** Quartile 1**	64 683	Reference (1)	Reference (0)	Reference (1)	Reference (0)	Reference (1)	Reference (0)
** Quartile 2**	64 690	**0.88 (0.81, 0.96)^b^**	**0.57 (0.16, 0.98)^b^**	**0.85 (0.75, 0.97)^b^**	**0.67 (0.13, 1.22)^b^**	**0.81 (0.67, 0.98)^b^**	**0.80 (0.70, 1.54)^b^**
** Quartile 3**	64 677	**0.83 (0.76, 0.92)^b^**	**0.89 (0.47, 1.32)^b^**	**0.79 (0.68, 0.91)^b^**	**1.00 (0.39, 1.60)^b^**	**0.75 (0.61, 0.92)^b^**	**1.09 (0.30, 1.87)^b^**
** Quartile 4**	64 682	**0.73 (0.66, 0.82)^b^**	**1.31 (0.81, 1.80)^b^**	**0.78 (0.66, 0.91)^b^**	**1.07 (0.40, 1.74)^b^**	**0.56 (0.43, 0.72)^b^**	**2.24 (1.24, 3.25)^b^**
**Normoglycemia**							
**Continuous**	191 946	**0.96 (0.94, 0.97)^b^**	**0.20 (0.11, 0.29)^b^**	**0.95 (0.92, 0.98)^b^**	**0.16 (0.04, 0.28)^b^**	**0.90 (0.85, 0.94)^b^**	**0.43 (0.24, 0.62)^b^**
**Categorical**							
** Quartile 1**	40 228	Reference (1)	Reference (0)	Reference (1)	Reference (0)	Reference (1)	Reference (0)
** Quartile 2**	49 077	**0.88 (0.79, 0.97)^b^**	**0.55 (0.07, 1.03)^b^**	**0.85 (0.73, 0.99)^b^**	**0.70 (0.04, 1.35)^b^**	0.80 (0.64, 1.01)	**0.94 (0.01, 1.87)^b^**
** Quartile 3**	49 631	**0.81 (0.72, 0.91)^b^**	**0.90 (0.38, 1.41)^b^**	**0.77 (0.65, 0.92)^b^**	**1.08 (0.29, 1.87)^b^**	**0.67 (0.52, 0.87)^b^**	**1.65 (1.63, 2.66)^b^**
** Quartile 4**	53 010	**0.75 (0.67, 0.86)^b^**	**1.13 (0.56, 1.70)^b^**	**0.78 (0.65, 0.94)^b^**	**1.13 (0.39, 1.86)^b^**	**0.54 (0.40, 0.73)^b^**	**2.59 (1.34, 3.85)^b^**
**Pre-diabetes**							
**Continuous**	66 786	**0.94 (0.91, 0.98)^b^**	**0.27 (0.12, 0.43)^b^**	0.96 (0.92, 1.01)	0.18 (−0.04, 0.40)	**0.93 (0.86, 0.99)^b^**	**0.32 (0.01, 0.64)^b^**
**Categorical**							
** Quartile 1**	24 455	Reference (1)	Reference (0)	Reference (1)	Reference (0)	Reference (1)	Reference (0)
** Quartile 2**	15 613	0.91 (0.78, 1.07)	0.48 (−0.31, 1.26)	0.86 (0.68, 1.09)	0.70 (−0.35, 1.75)	0.84 (0.60, 1.18)	0.72 (−0.74, 2.19)
** Quartile 3**	15 046	0.89 (0.76, 1.06)	0.49 (−0.27, 1.25)	0.83 (0.65, 1.07)	0.82 (−0.30, 1.94)	0.93 (0.66, 1.32)	0.29 (−1.16, 1.74)
** Quartile 4**	11 672	**0.69 (0.55, 0.86)^b^**	**1.80 (0.76, 2.84)^b^**	**0.75 (0.55, 0.96)^b^**	**1.32 (0.08, 2.73)^b^**	**0.52 (0.31, 0.89)^b^**	**2.69 (0.47, 4.90)^b^**

a All models were adjusted for age, sex, education, socioeconomic status, body mass index, smoking status, alcohol drinking, physical activity, social contact, dyslipidemia, cardiovascular disease, and *apolipoprotein* E ε4.

b *p *< .05.

Bold values indicate statistically significant differences.

Laplace regression indicated that participants in higher eGDR quartiles experienced later dementia onset. Compared to quartile 1, dementia was delayed by 0.57 years in quartile 2, 0.89 years in quartile 3, and 1.31 years in quartile 4. Specifically, the onset was delayed by 1.07 (eGDR quartile 4) years for AD and 2.24 years for VaD, respectively (Table 2).

Stratified by glycemic status, the inverse association between eGDR and dementia risk remained significant in normoglycemic and prediabetic subgroups, with no significant interaction (Table 2).

### Association between eGDR and brain age gap

Higher eGDR was dose-dependently related to smaller BAG (β: −0.33 [−0.38, −0.27]; Table 3), as well as among people with normoglycemia (−0.37 [−0.44, −0.29]) and prediabetes (−0.29 [−0.37, −0.22]). Compared to the lowest eGDR quartile, the β (95% CI) of quartiles 2-4 were −0.98 (−1.31, −0.65), −1.08 (−1.41, −0.75), and −2.09 (−2.45, −1.74) for BAG (Table 3). The associations between higher eGDR and smaller BAG were consistent among participants with different glycemic status (*p*  _for interaction_ > .05; Table 3). The distribution of BAG in each eGDR group is shown in Supplementary Figure S3.

**Table 3.** Standardized β coefficient and 95% confidence interval (CI) for the association between estimated glucose disposal rate (eGDR) and brain age gap (BAG): results from linear regressions.

eGDR	No. of participants	LS Mean ± SE^b^	BAG			
			β (95% CI)^a^	*p*-value	β (95% CI)^b^	*p*-Value
**All participants**						
**Continuous**	15 389	0.13 ± 0.05	−0.34 (−0.38, −0.30)	<.001	−0.33 (−0.38, −0.27)	<.001
**Categorical**						
** Quartile 1**	2834	1.37 ± 0.11	Reference (0)	−	Reference (0)	−
** Quartile 2**	3474	0.33 ± 0.10	−0.92 (−1.19, −0.66)	<.001	−0.98 (−1.31, −0.65)	<.001
** Quartile 3**	4037	0.26 ± 0.09	−1.21 (−1.47, −0.95)	<.001	−1.08 (−1.41, −0.75)	<.001
** Quartile 4**	5044	−0.78 ± 0.08	−2.13 (−2.39, −1.87)	<.001	−2.09 (−2.45, −1.74)	<.001
**Normoglycemia**						
**Continuous**	12 194	0.04 ± 0.05	−0.34 (−0.38, −0.29)	<.001	−0.37 (−0.44, −0.29)	<.001
**Categorical**						
** Quartile 1**	1926	1.30 ± 0.12	Reference (0)	−	Reference (0)	-
** Quartile 2**	2779	0.30 ± 0.10	−0.80 (−1.10, −0.50)	<.001	−1.20 (−1.69, −0.71)	<.001
** Quartile 3**	3223	0.22 ± 0.10	−1.15 (−1.45, −0.85)	<.001	−1.51 (−2.01, −1.01)	<.001
** Quartile 4**	4266	−0.81 ± 0.09	−2.08 (−2.38, −1.79)	<.001	−2.49 (−3.01, −1.97)	<.001
**Pre-diabetes**						
**Continuous**	3195	0.46 ± 0.11	−0.32 (−0.42, −0.22)	<.001	−0.29 (−0.37, −0.22)	<.001
**Categorical**						
** Quartile 1**	908	1.51 ± 0.14	Reference (0)	−	Reference (0)	−
** Quartile 2**	695	0.46 ± 0.14	−1.03 (−1.61, −0.44)	.001	−0.84 (−1.29, −0.38)	<.001
** Quartile 3**	814	0.42 ± 0.14	−1.27 (−1.82, −0.72)	<.001	−0.81 (−1.25, −0.37)	<.001
** Quartile 4**	778	−0.62 ± 0.13	−1.93 (−2.53, −1.32)	<.001	−1.81 (−2.30, −1.32)	<.001

a Models were adjusted for age, sex, and education.

b Further adjusted for socioeconomic status, body mass index, smoking status, alcohol drinking, physical activity, social contact, dyslipidemia, cardiovascular disease, and *apolipoprotein* E ε4.

### Role of sex, physical activity, BMI, and genetic background

**Figure 2.**
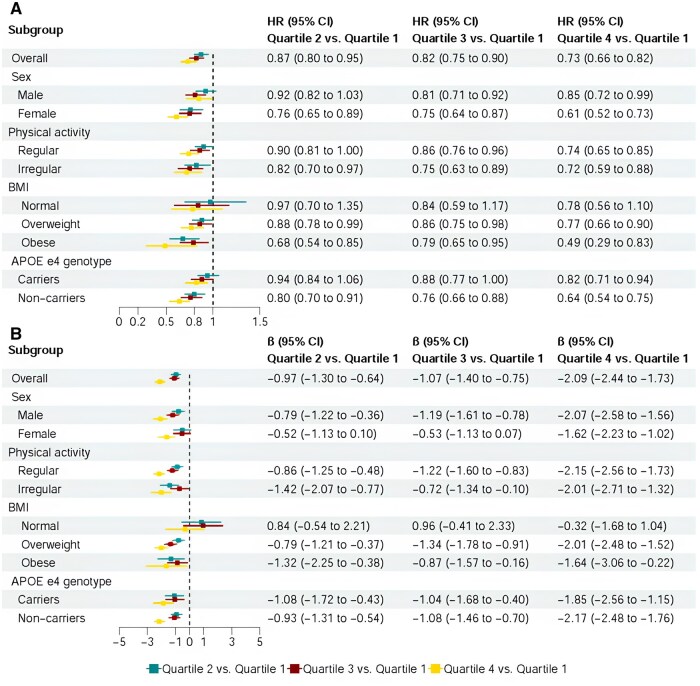
The association of estimated glucose disposal rate (eGDR) with dementia and brain age gap (BAG): stratified by sex, physical activity, body mass index (BMI), and *APOE* ɛ4. Panel A. Dementia risk: significant multiplicative interactions were detected between eGDR and sex/*APOE* ɛ4 on dementia (*p*  _for interaction_ < .05). Panel B. BAG: Significant multiplicative interactions were detected between eGDR and sex/BMI (*p*  _for interaction_ < .05). All models were adjusted for age, sex, education, socioeconomic status, body mass index, smoking status, alcohol drinking, physical activity, social activity, dyslipidemia, pre-diabetes, and cardiovascular disease (if applicable).

In stratified analyses (Figure 2A), the association between eGDR and dementia was stronger in females versus males and in *APOE* ɛ4 noncarriers versus carriers. Significant interactions were detected between eGDR and sex/*APOE* ɛ4 concerning dementia onset (all *p*  _for interaction_ < .05). No significant interactions were found between eGDR and physical activity or BMI.

For BAG, the association with eGDR was stronger in men and among overweight/obese participants, with significant interactions for sex and BMI (Figure 2B). No significant interactions were found between eGDR and physical activity or *APOE* ɛ4.

In joint-effect analysis, high eGDR (quartile 4) significantly attenuated the association between *APOE* ɛ4 and dementia/BAG. Among *APOE* ε4 carriers, those in eGDR quartile 4 had a 14% lower dementia risk (HR: 0.86, 0.76-0.98) and a BAG that was 1.77 years younger (β: −1.77, −2.34-−1.19), compared to carriers in eGDR quartile 1 (Supplementary Figure S2 and Supplementary Tables S4 and S5). In addition, *APOE* ɛ4 noncarriers with eGDR quartile 4 have the lowest dementia risk and the lowest BAG.

### Sensitivity analysis

Similar results were yielded (Supplementary Tables S6-S9) when we repeated the analyses after (1) performing basic-adjusted models with age, sex, and education as covariates; (2) performing competing risk models with death as competing events (competing events = 25 031); (3) excluding 183 incident dementia cases that occurred during the first 3 years of the follow-up; and (4) performing multiple imputation for missing values of covariates.

## Discussion

In this large-scale cohort study of older adults without diabetes and dementia, we found that high eGDR—as estimated by waist circumference, hypertension, and HbA1c—is associated with a 27% lower risk of dementia, a 1.31-year delay in the onset of dementia, and a brain age that is 2.09 years younger than expected chronological age. The associations between low eGDR and higher dementia risk and older brain age were present among participants with both normoglycemia and pre-diabetes alike. Additionally, high eGDR may attenuate the *APOE* ε4 genetic risk of dementia and brain aging among diabetes-free people.

Accumulating evidence has linked IR to cognitive decline^33^ and the risk of dementia.^13^^,^^34^ In a recent meta-analysis of three cohort studies, another surrogate marker of IR, the TyG index, was associated with an increased risk of dementia.^34^ A nationwide observational cohort study in Korea also found that TyG was associated with AD and VaD.^7^ A follow-up survey of 1544 diabetes- and dementia-free older men in Japan showed that IR, measured by the McAuley and HOMA indices, was associated with dementia and AD.^13^ However, a cohort from the Framingham Heart Study found no significant association between glucose homeostasis biomarkers (adiponectin, glucose, glycated albumin, and insulin levels) and dementia.^35^ Although these prior studies provide evidence about the possible impact of IR on dementia, most of them did not consider the effects of various glycemic levels. In addition, the complexity of the McAuley and HOMA indices makes them difficult to apply to large-scale population studies, and the TyG index’s sensitivity may not be sufficient, which limits the generalizability of the findings from previous research.^17^^,^^18^

Accumulating evidence suggests that a significantly increased risk of dementia also emerges in people with pre-diabetes.^10^^,^^11^ However, whether IR plays a role in the process is unknown. Our findings suggest that increased IR is associated with an increased risk of dementia in people with pre-diabetes, and even in those with normoglycemia, which indicates that the adverse effect of IR on cognitive aging occurs even before the onset of diabetes. Thus, IR could serve as an earlier predictor of dementia risk than diabetes itself. In addition, we found that the eGDR-dementia association was more pronounced among females and *APOE* ε4 noncarriers. These findings may suggest that interventions targeting IR to prevent dementia could be more impactful in females and *APOE* ε4 noncarriers, and are nonetheless important in all groups, but this needs to be addressed in more depth in future studies.

To complement these findings, we explored the association between eGDR and BAG assessed by multimodal brain MRI metrics. Individuals with high eGDR had significantly younger brain ages, and similarly, low IR could also buffer the brain aging in relation to *APOE* ε4 genetic risk. Although previous studies have also linked IR to markers of poorer brain health (including smaller gray matter volumes,^14^ larger periventricular hyperintensities, and larger volumes of deep and subcortical white matter hyperintensities^15^) they have not fully captured the extent of individual brain aging. In light of the limited evidence on the association between IR and individual MRI phenotypes, our findings provide convincing evidence that IR may accelerate brain aging even at the preclinical stage of neurodegenerative disease.

### Potential mechanisms

Several possible mechanisms could underlie the association of IR with dementia and brain aging in people without diabetes. On the one hand, cerebral IR can impair insulin activity and glucose metabolism, exacerbating neuronal cell death,^36^ which in turn causes cognitive impairment and dementia.^37^^,^^38^ On the other hand, IR is associated with a variety of cerebrovascular diseases, which may result in multifocal ischemic lesions that affect brain function.^39^ In addition, insulin could regulate peripheral and cerebral blood flow, and disruptions in insulin-related vasodilation may lead to cerebral hypoperfusion and ischemic lesions.^40^ In turn, cerebral ischemic lesions are directly related to the development of AD and VaD.^41^ Further experimental studies are required to clarify the mechanisms underlying the association between IR and dementia.

### Strengths and limitations

Strengths of this study include the community-based design with a large sample size and a rigorous data collection procedure. Additionally, we were able to consider a wide range of confounders by leveraging the extensive information available in the UK Biobank. Nonetheless, some limitations should be pointed out. First, UK Biobank participants were volunteers, potentially representing a healthier subset of the general population.^42^^,^^43^ Moreover, our analytical sample consisted of participants who underwent brain MRI scans and were free from chronic brain disorders, which is a relatively healthier subset in the overall UK Biobank population. Consequently, this may have led to an underestimation of the association of high IR with increased dementia risk and accelerated brain aging. Furthermore, caution is required when generalizing our results to populations outside of white European ancestry. Second, the use of electronic health records for diagnosis may have high specificity but low sensitivity,^44^ and some mild dementia cases may not have been captured, which may attenuate the relationship between eGDR and dementia risk. Third, dementia has a decades-long preclinical phase, and the calculation of eGDR may be biased among participants with preclinical or prodromal dementia. To address this, we conducted a sensitivity analysis excluding participants with likely preclinical/prodromal dementia (ie, incident dementia cases that occurred during the first 3 years of follow-up). Despite this exclusion, the eGDR-dementia association persisted. Fourth, although eGDR provides a more practical and feasible alternative to the gold standard technique, future studies should compare the effects of eGDR with clamp-measured insulin sensitivity in dementia risk prediction to further clarify its biological significance. Finally, selection bias might have occurred due to missing data. However, the results were not much altered after repeating the analysis using multiple imputations for missing variables.

## Conclusion

In conclusion, our study provides valuable insights that eGDR can predict the onset of dementia and accelerated brain aging, even among people with normoglycemia and pre-diabetes. Therefore, eGDR might serve as a modifiable preclinical predictor of dementia among older adults. Our findings highlight IR as a potential indicator of brain health among older adults across the glycemic spectrum.

## Supplementary Material

glaf243_Supplementary_Data

## Data Availability

The datasets generated and analyzed during the current study are available in the UK Biobank repository, http://www.ukbiobank.ac.uk.
