# Supplementary material for: Estimated glucose disposal rate is associated with brain aging and dementia among diabetes-free older adults
Source: J Gerontol A Biol Sci Med Sci. 2025 Oct 31;81(1):glaf243. doi: 10.1093/gerona/glaf243 (PMC12758966; doi:10.1093/gerona/glaf243)
Supplement: glaf243_Supplementary_Data [file glaf243_supplementary_data.zip › Supplementary materials.docx]

**Baseline included participants, n=502,353**

We excluded 207,585 persons due to:

- - Prevalent dementia (n=573)
  - Baseline age<55 (n=94,725)
  - Missing information for eGDR calculation (n=36,423)

**Analytical sample, n=258,732**

**Follow-up time:** 3607.4 (×1000 person-years**)**

**Incident dementia:** 7,063 (2.73%)

We excluded 36,036 persons with prevalent diabetes (including type 1 and type 2).

**Diabetes-free participants, n=466,317**

**Neuroimaging subsample:**

15,389 chronic brain disorders-free persons

**Supplementary Figure 1.** Flowchart of the study population.

**Supplementary Table 1.** Self-reported health variables codes used for exclusion criteria on the initial population.

| **Self-reported illness** | **Code (Field ID 20002 and 20003)** |
| --- | --- |
| Dementia or Alzheimer’s disease | 1263 |
| Parkinson’s disease | 1262 |
| Chronic degenerative neurological | 1258 |
| Guillain-Barré syndrome | 1256 |
| Multiple Sclerosis | 1261 |
| Other demyelinating disease | 1397 |
| Stroke or ischemic stroke | 1081 |
| Brain cancer | 1032 |
| Brain hemorrhage | 1491 |
| Brain/intracranial abscess | 1245 |
| Cerebral aneurysm | 1425 |
| Cerebral palsy | 1433 |
| Encephalitis | 1246 |
| Epilepsy | 1264 |
| Head injury | 1266 |
| Infections of the nervous system | 1244 |
| Ischemic stroke | 1583 |
| Meningeal cancer | 1031 |
| Meningioma (benign) | 1659 |
| Meningitis | 1247 |
| Motor Neuron Disease | 1259 |
| Neurological injury/trauma | 1240 |
| Spina bifida | 1524 |
| Subdural hematoma | 1083 |
| Subarachnoid hemorrhage | 1086 |
| Transient ischemic attack | 1082 |

**Supplementary Table 2.** International Classification of Diseases (ICD) code list for dementia.

| **ICD Codes** | **Text** | **Alzheimer’s disease** | **Vascular dementia** | **All-cause Dementia** |
| --- | --- | --- | --- | --- |
| **ICD-9** |  |  |  |  |
| 290.2 | Senile dementia, depressed or paranoid type |  |  | 🗸 |
| 290.3 | Senile dementia with acute confusional state |  |  | 🗸 |
| 290.4 | Arteriosclerotic dementia |  | 🗸 | 🗸 |
| 291.2 | Other alcoholic dementia |  |  | 🗸 |
| 294.1 | Dementia in other conditions classified elsewhere |  |  | 🗸 |
| 331.0 | Alzheimer's disease | 🗸 |  | 🗸 |
| 331.1 | Pick's disease |  |  | 🗸 |
| 331.2 | Senile degeneration of brain |  |  | 🗸 |
| 331.5 | Creutzfeldt-Jakob disease |  |  | 🗸 |
| **ICD-10** |  |  |  |  |
| A81.0 | Sporadic Creutzfeldt-Jakob disease |  |  | 🗸 |
| F00 | Dementia in Alzheimer's disease | 🗸 |  | 🗸 |
| F00.0 | Dementia in Alzheimer's disease with early onset | 🗸 |  | 🗸 |
| F00.1 | Dementia in Alzheimer's disease with late onset | 🗸 |  | 🗸 |
| F00.2 | Dementia in Alzheimer's disease, atypical or mixed type | 🗸 |  | 🗸 |
| F00.9 | Dementia in Alzheimer's disease, unspecified | 🗸 |  | 🗸 |
| F01 | Vascular dementia |  | 🗸 | 🗸 |
| F01.0 | Vascular dementia of acute onset |  | 🗸 | 🗸 |
| F01.1 | Multi-infarct dementia |  | 🗸 | 🗸 |
| F01.2 | Subcortical vascular dementia |  | 🗸 | 🗸 |
| F01.3 | Mixed cortical and sub-cortical vascular dementia |  | 🗸 | 🗸 |
| F01.8 | Other vascular dementia |  | 🗸 | 🗸 |
| F01.9 | Vascular dementia, unspecified |  | 🗸 | 🗸 |
| F02 | Dementia in other diseases classified elsewhere |  |  | 🗸 |
| F02.0 | Dementia in Picks disease |  |  | 🗸 |
| F02.1 | Dementia in Creutzfeldt-Jacob disease |  |  | 🗸 |
| F02.2 | Dementia in Huntington’s disease |  |  | 🗸 |
| F02.3 | Dementia in Parkinson’s disease |  |  | 🗸 |
| F02.4 | Dementia in HIV disease |  |  | 🗸 |
| F02.8 | Dementia in other specified diseases classified elsewhere |  |  | 🗸 |
| F03 | Unspecified dementia |  |  | 🗸 |
| F05.1 | Delirium superimposed on dementia |  |  | 🗸 |
| F10.6 | Mental and behavioural disorders due to use of alcohol-amnesic syndrome |  |  | 🗸 |
| G30 | Alzheimer’s disease | 🗸 |  | 🗸 |
| G30.0 | Alzheimer’s disease with early onset | 🗸 |  | 🗸 |
| G30.1 | Alzheimer’s disease with late onset | 🗸 |  | 🗸 |
| G30.8 | Other Alzheimer's disease | 🗸 |  | 🗸 |
| G30.9 | Alzheimer's disease unspecified | 🗸 |  | 🗸 |
| G31.0 | Circumscribed brain atrophy |  |  | 🗸 |
| G31.1 | Senile degeneration of brain |  |  | 🗸 |
| G31.8 | Other specified degenerative diseases of nervous system |  |  | 🗸 |
| I67.3 | Binswanger’s disease |  | 🗸 |  |

**Supplementary Table 3.** Baseline characteristics of the neuroimaging subsamples* by estimated glucose disposal rate (eGDR) (N= 15,389).

| Characteristics | eGDR | | | | *P* value |
| --- | --- | --- | --- | --- | --- |
|  | Quartile 1 (n = 2,834) | Quartile 2 (n = 3,474) | Quartile 3  (n = 4,037) | Quartile 4  (n = 5,044) |  |
| Follow-up time (year) | 14.51  (13.73, 15.23) | 14.59  (13.88, 15.24) | 14.62  (13.93, 15.28) | 14.67  (13.97, 15.35) | - |
| eGDR (mg/kg/min) | 5.44 ± 0.64 | 6.77 ± 0.36 | 8.69 ± 0.80 | 11.04 ± 0.67 | <0.001 |
| Age (year) | 61.18 ± 3.86 | 61.45 ± 3.88 | 60.70 ± 3.76 | 59.98 ± 3.67 | <0.001 |
| Female | 587 (20.71) | 1410 (40.59) | 2207 (54.67) | 3593 (71.23) | <0.001 |
| Townsend deprivation index | -2.72  (-3.96, -0.80) | -2.82  (-3.98, -1.09) | -2.81  (-3.94, -0.97) | -2.77  (-3.97, -0.91) | 0.258 |
| Education (college) | 1104 (39.20) | 1509 (43.56) | 1726 (42.88) | 2457 (48.88) | <0.001 |
| BMI (kg/m^2^) | 30.46 ± 3.67 | 26.59 ± 2.66 | 26.14 ± 3.73 | 24.23 ± 2.61 | <0.001 |
| Underweight (<20) | 0 (0.00) | 5 (0.14) | 113 (2.80) | 211 (4.19) | <0.001 |
| Normal (20 – 25) | 66 (2.33) | 953 (27.44) | 1561 (38.68) | 3028 (60.07) |  |
| Overweight (25 – 30) | 1398 (49.42) | 2184 (62.89) | 1793 (44.43) | 1686 (33.45) |  |
| Obese (≥30) | 1365 (48.25) | 331 (9.53) | 569 (14.10) | 116 (2.30) |  |
| Alcohol drinking |  |  |  |  | 0.022 |
| Never | 52 (1.84) | 65 (1.87) | 87 (2.16) | 147 (2.92) |  |
| Former drinker | 55 (1.94) | 66 (1.90) | 76 (1.88) | 101 (2.00) |  |
| Current drinker | 2726 (96.22) | 3341 (96.23) | 3873 (95.96) | 4794 (95.08) |  |
| Smoking status |  |  |  |  | <0.001 |
| Never | 1409 (49.84) | 1961 (56.58) | 2320 (57.70) | 3107 (61.67) |  |
| Former smoker | 1276 (45.14) | 1358 (39.18) | 1493 (37.13) | 1678 (33.31) |  |
| Current smoker | 142 (5.02) | 147 (4.24) | 208 (5.17) | 253 (5.02) |  |
| Regular physical activity | 1847 (68.18) | 2584 (77.07) | 2932 (75.53) | 3809 (78.29) | <0.001 |
| High level of social contact | 1605 (56.81) | 2011 (58.10) | 2228 (55.34) | 2730 (54.22) | 0.003 |
| Dyslipidemia | 1416 (52.44) | 1620 (48.96) | 1905 (49.67) | 2237 (46.20) | <0.001 |
| TC (mmol/L) | 5.63 ± 1.15 | 5.86 ± 1.16 | 5.97 ± 1.06 | 6.01 ± 1.03 | <0.001 |
| LDL-C (mmol/L) | 3.59 ± 0.88 | 3.69 ± 0.88 | 3.73 ± 0.81 | 3.72 ± 0.79 | <0.001 |
| TG (mmol/L) | 2.09 ± 1.05 | 1.74 ± 0.90 | 1.66 ± 0.91 | 1.42 ± 0.73 | <0.001 |
| Cardiovascular disease | 255 (9.00) | 255 (7.34) | 174 (4.31) | 105 (2.08) | <0.001 |
| Pre-diabetes | 908 (32.04) | 695 (20.01) | 814 (20.16) | 778 (15.42) | <0.001 |
| *APOE* ɛ4 carriers | 641 (27.00) | 793 (27.08) | 894 (26.06) | 1177 (26.97) | 0.760 |

Abbreviations: BMI = body mass index; TC = Total cholesterol; LDL-C = low-density lipoprotein cholesterol; TG = total triglyceride; *APOE* = *apolipoprotein* E.

* The neuroimaging samples are a healthier sub-sample of the entire cohort with younger age, healthier lifestyle, and a lower proportion of chronic disease.


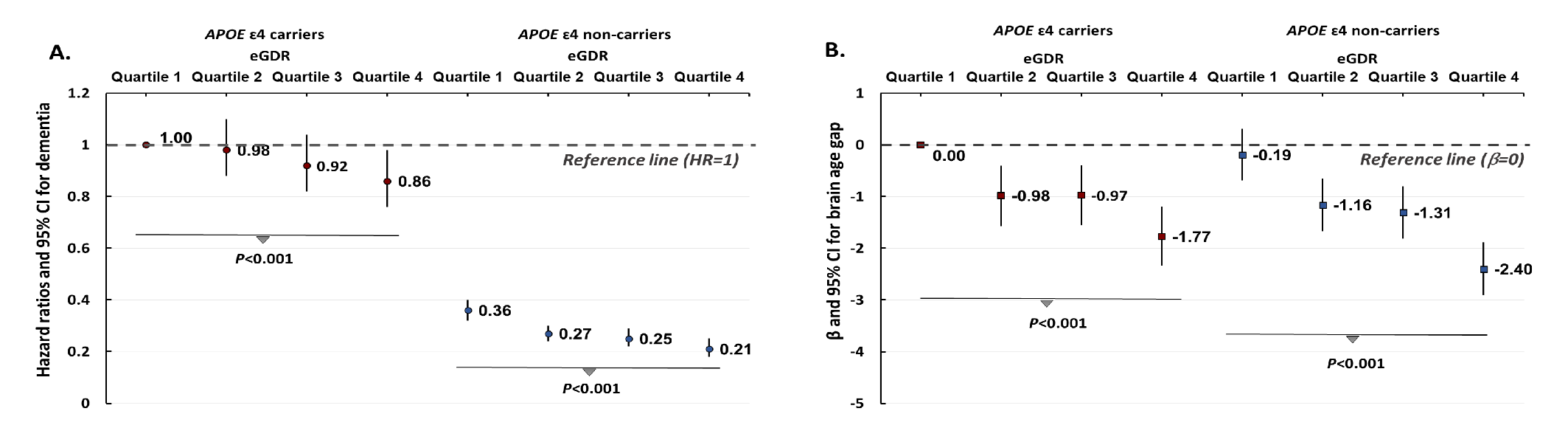


**Supplementary Figure 2.** Joint effect of estimated glucose disposal rate (eGDR) and APOE ɛ4 on incident dementia and brain age gap (BAG).

Panel A. Dementia risk was assessed using Cox regressions. A significant interaction was detected between eGDR and APOE ɛ4 (P <0.001). Panel B. BAG was evaluated using linear regressions. Models were adjusted for age, sex, education, socioeconomic status, body mass index, smoking status, alcohol drinking, physical activity, social activity, dyslipidemia, pre-diabetes, and cardiovascular disease. In all models, significant differences between the *APOE* ɛ4 non-carriers with eGDR Quartile 1 and *APOE* ɛ4 non-carriers with eGDR Quartile 4 groups were assessed by repeating the models using the *APOE* ɛ4 non-carriers with eGDR Quartile 1 group as the reference. Also, significant differences between the *APOE* ɛ4 carriers with eGDR Quartile 1 and *APOE* ɛ4 carriers with eGDR Quartile 4 groups were assessed by repeating the models using the *APOE* ɛ4 carriers with eGDR Quartile 1 group as the reference.

**Supplementary Table 4.** Hazard ratios (HRs) and 10th percentile differences (PDs) in years of onset for incident dementia in relation to estimated glucose disposal rate (eGDR)

| **Joint exposure** | | ***No. of participants*** | ***No. of cases*** | **Dementia** |
| --- | --- | --- | --- | --- |
| ***APOE* ɛ4** | **eGDR** |  |  | HR (95% CI) ^a^ |
| Carriers | Quartile 1 | 14,940 | 869 | Reference (1) |
|  | Quartile 2 | 15,363 | 938 | 0.98 (0.88, 1.10) |
|  | Quartile 3 | 15,636 | 846 | 0.92 (0.82, 1.04) |
|  | Quartile 4 | 15,827 | 664 | 0.86 (0.76, 0.98) ^b^ |
| Non-carriers | Quartile 1 | 38,354 | 862 | 0.36 (0.32, 0.40) ^b^ |
|  | Quartile 2 | 38,807 | 697 | 0.27 (0.24, 0.30) ^b^ |
|  | Quartile 3 | 38,881 | 612 | 0.25 (0.22, 0.29) ^b^ |
|  | Quartile 4 | 39,275 | 448 | 0.21 (0.76, 0.98) ^b^ |

^a^ All models were adjusted for age, sex, education, socioeconomic status, body mass index, smoking status, alcohol drinking, physical activity, social activity, dyslipidemia, and cardiovascular disease.

^b^ *P*< 0.05.


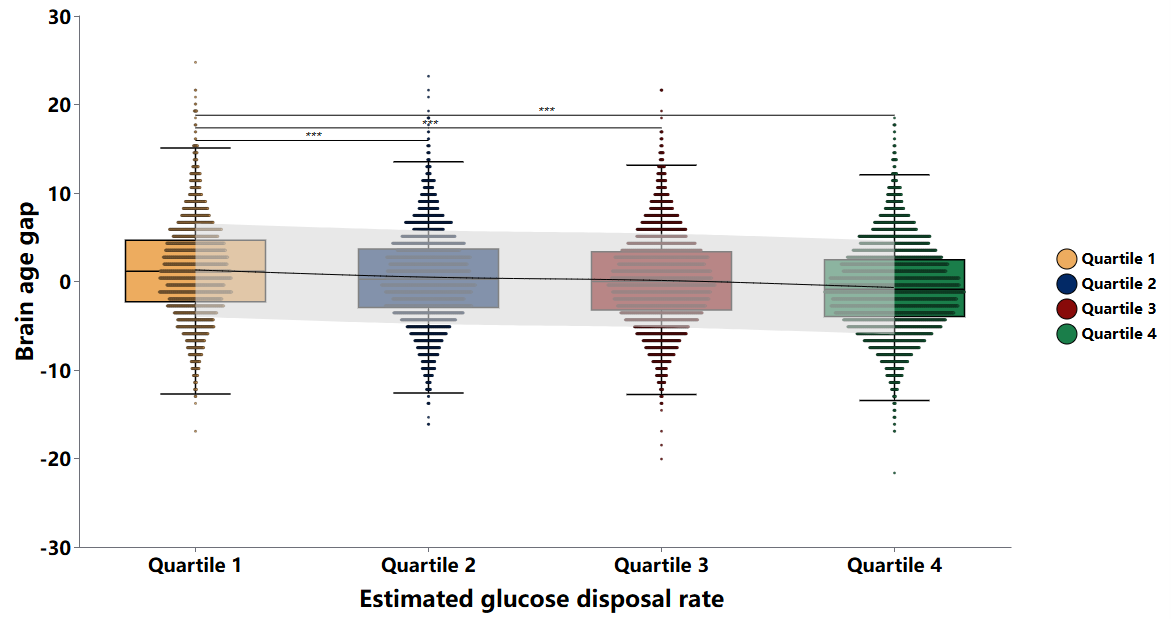


**Supplementary Figure 3.** The difference in brain age gap between different estimated glucose disposal rate (eGDR) groups.

^***^*P*<0.001. Compared to eGDR Quartile 1, the brain age gap in Quartile 2-4 was significantly smaller.

**Supplementary Table 5.** Standardized β coefficient and 95% confidence interval (CI) for the joint effect between estimated glucose disposal rate (eGDR) and *APOE* ɛ4 on brain age gap (BAG).

| **Joint exposure** | | ***No. of participants*** | ***LS Mean ± SE*** | **BAG** | |
| --- | --- | --- | --- | --- | --- |
| ***APOE* ɛ4** | **eGDR** |  |  | β (95% CI) ^a^ | P |
| Carriers | Quartile 1 | 641 | 1.53 ± 0.22 | Reference (0) | - |
|  | Quartile 2 | 793 | 0.48 ± 0.19 | -0.98 (-1.57, -0.40) ^b^ | 0.001 |
|  | Quartile 3 | 894 | 0.49 ± 0.18 | -0.97 (-1.55, -0.39) ^b^ | 0.001 |
|  | Quartile 4 | 1,177 | -0.31 ± 0.16 | -1.77 (-2.34, -1.19) ^b^ | <0.001 |
| Non-carriers | Quartile 1 | 1,733 | 1.30 ± 0.13 | -0.19 (-0.69, 0.31) | 0.449 |
|  | Quartile 2 | 2,135 | 0.28 ± 0.12 | -1.16 (-1.67, -0.65) ^b^ | <0.001 |
|  | Quartile 3 | 2,536 | 0.18 ± 0.11 | -1.31 (-1.81, -0.80) ^b^ | <0.001 |
|  | Quartile 4 | 3,187 | -0.95 ± 0.10 | -2.40 (-2.91, -1.88) ^b^ | <0.001 |

^a^ All models were adjusted for age, sex, education, socioeconomic status, body mass index, smoking status, alcohol drinking, physical activity, social activity, dyslipidemia, and cardiovascular disease.

^b^ *P*< 0.05.

**Supplementary Table 6.** Hazard ratios (HRs) and 10th percentile differences (PDs) in years of onset for incident dementia in relation to estimated glucose disposal rate (eGDR): basic-adjusted models.

| **eGDR** | ***No. of participants*** | **Dementia**  (cases= 7,063) | | **Dementia subtypes** | | | |
| --- | --- | --- | --- | --- | --- | --- | --- |
|  |  |  |  | **Alzheimer’s Disease**  (cases= 3,285) | | **Vascular Dementia**  (cases= 1,428) | |
|  |  | HR (95% CI) ^a^ | 10th PD (95% CI) ^a^ | HR (95% CI) ^a^ | 10th PD (95% CI) ^a^ | HR (95% CI) ^a^ | 10th PD (95% CI) ^a^ |
| Continuous | 258,732 | 0.97 (0.97, 0.98) ^b^ | 0.16 (0.10, 0.21) ^b^ | 0.99 (0.98, 1.01) | 0.03 (-0.04, 0.11) | 0.90 (0.88, 0.93) ^b^ | 0.44 (0.32, 0.56) ^b^ |
| Categorical |  |  |  |  |  |  |  |
| Quartile 1 | 64,683 | Reference (1) | Reference (0) | Reference (1) | Reference (0) | Reference (1) | Reference (0) |
| Quartile 2 | 64,690 | 0.92 (0.86, 0.98) ^b^ | 0.43 (0.13, 0.72) ^b^ | 0.96 (0.87, 1.06) | 0.19 (-0.20, 0.58) | 0.79 (0.69, 0.90) ^b^ | 0.94 (0.40, 1.48) ^b^ |
| Quartile 3 | 64,677 | 0.91 (0.85, 0.97) ^b^ | 0.48 (0.17, 0.80) ^b^ | 0.95 (0.86, 1.06) | 0.22 (-0.19, 0.62) | 0.73 (0.63, 0.85) ^b^ | 1.28 (0.69, 1.86) ^b^ |
| Quartile 4 | 64,682 | 0.81 (0.75, 0.88) ^b^ | 0.99 (0.64, 1.35) ^b^ | 0.96 (0.86, 1.07) | 0.24 (-0.21, 0.69) | 0.54 (0.45, 0.64) ^b^ | 2.48 (1.75, 3.21) ^b^ |

^a^ All models were adjusted for age, sex, and education.

^b^ *P*< 0.05.

**Supplementary Table 7.** Hazard ratios (HRs) and 10th percentile differences (PDs) in years of onset for incident dementia in relation to estimated glucose disposal rate (eGDR): after excluding incident dementia cases during the first 3 years of follow-up.

| **eGDR** | ***No. of participants*** | **Dementia**  (cases= 6,880) | | **Dementia subtypes** | | | |
| --- | --- | --- | --- | --- | --- | --- | --- |
|  |  |  |  | **Alzheimer’s Disease**  (cases= 3,214) | | **Vascular Dementia**  (cases= 1,390) | |
|  |  | HR (95% CI) ^a^ | 10th PD (95% CI) ^a^ | HR (95% CI) ^a^ | 10th PD (95% CI) ^a^ | HR (95% CI) ^a^ | 10th PD (95% CI) ^a^ |
| Continuous | 255,954 | 0.95 (0.93, 0.97) ^b^ | 0.24 (0.16, 0.31) ^b^ | 0.96 (0.94, 0.99) ^b^ | 0.18 (0.07, 0.28) ^b^ | 0.90 (0.87, 0.94) ^b^ | 0.41 (0.25, 0.58) ^b^ |
| Categorical |  |  |  |  |  |  |  |
| Quartile 1 | 63,754 | Reference (1) | Reference (0) | Reference (1) | Reference (0) | Reference (1) | Reference (0) |
| Quartile 2 | 63,990 | 0.86 (0.79, 0.94) ^b^ | 0.64 (0.24, 1.04) ^b^ | 0.83 (0.73, 0.95) ^b^ | 0.79 (0.26, 1.32) ^b^ | 0.80 (0.66, 0.97) ^b^ | 0.88 (0.14, 1.63) ^b^ |
| Quartile 3 | 64,038 | 0.80 (0.72, 0.88) ^b^ | 1.01 (0.59, 1.42) ^b^ | 0.76 (0.66, 0.87) ^b^ | 1.15 (0.56, 1.76) ^b^ | 0.72 (0.58, 0.89) ^b^ | 1.34 (0.53, 2.15) ^b^ |
| Quartile 4 | 64,172 | 0.73 (0.65, 0.82) ^b^ | 1.37 (0.89, 1.85) ^b^ | 0.75 (0.64, 0.88) ^b^ | 1.23 (0.57, 1.89) ^b^ | 0.55 (0.43, 0.72) ^b^ | 2.42 (1.37, 3.46) ^b^ |

^a^ All models were adjusted for age, sex, education, socioeconomic status, body mass index, smoking status, alcohol drinking, physical activity, social activity, dyslipidemia, cardiovascular disease, pre-diabetes, and *apolipoprotein* E ɛ4.

^b^ *P*< 0.05.

**Supplementary Table 8.** Hazard ratios (HRs) and 10th percentile differences (PDs) in years of onset for incident dementia in relation to estimated glucose disposal rate (eGDR): using a competing risk model with death as competing events.

| **eGDR** | ***No. of participants*** | ***No. of death*** | **Dementia**  (cases= 7,063) | **Dementia subtypes** | |
| --- | --- | --- | --- | --- | --- |
|  |  |  |  | **Alzheimer’s Disease**  (cases= 3,285) | **Vascular Dementia**  (cases= 1,428) |
|  |  |  | HR (95% CI) ^a^ | HR (95% CI) ^a^ | HR (95% CI) ^a^ |
| Continuous | 258,732 | 28,608 | 0.94 (0.93, 0.95) ^b^ | 0.94 (0.93, 0.95) ^b^ | 0.95 (0.94, 0.96) ^b^ |
| Categorical |  |  |  |  |  |
| Quartile 1 | 64,683 | 10,058 | Reference (1) | Reference (1) | Reference (1) |
| Quartile 2 | 64,690 | 8,962 | 0.86 (0.82, 0.90) ^b^ | 0.86 (0.82, 0.90) ^b^ | 0.86 (0.82, 0.89) ^b^ |
| Quartile 3 | 64,677 | 5,936 | 0.79 (0.75, 0.83) ^b^ | 0.80 (0.76, 0.83) ^b^ | 0.80 (0.76, 0.84) ^b^ |
| Quartile 4 | 64,682 | 3,652 | 0.75 (0.70, 0.80) ^b^ | 0.74 (0.70, 0.79) ^b^ | 0.75 (0.71, 0.80) ^b^ |

^a^ All models were adjusted for age, sex, education, socioeconomic status, body mass index, smoking status, alcohol drinking, physical activity, social activity, dyslipidemia, cardiovascular disease, pre-diabetes, and *apolipoprotein* E ɛ4.

^b^ *P*< 0.05.

**Supplementary Table 9.** Hazard ratios (HRs) and 10th percentile differences (PDs) in years of onset for incident dementia in relation to estimated glucose disposal rate (eGDR): after performing multiple imputation of missing values for covariates.

| **eGDR** | ***No. of participants*** | **Dementia**  (cases= 6,880) | | **Dementia subtypes** | | | |
| --- | --- | --- | --- | --- | --- | --- | --- |
|  |  |  |  | **Alzheimer’s Disease**  (cases= 3,214) | | **Vascular Dementia**  (cases= 1,390) | |
|  |  | HR (95% CI) ^a^ | 10th PD (95% CI) ^a^ | HR (95% CI) ^a^ | 10th PD (95% CI) ^a^ | HR (95% CI) ^a^ | 10th PD (95% CI) ^a^ |
| Continuous | 255,954 | 0.96 (0.94, 0.97) ^b^ | 0.21 (0.14, 0.29) ^b^ | 0.96 (0.94, 0.98) ^b^ | 0.17 (0.07, 0.27) ^b^ | 0.91 (0.88, 0.95) ^b^ | 0.39 (0.24, 0.55) ^b^ |
| Categorical |  |  |  |  |  |  |  |
| Quartile 1 | 63,754 | Reference (1) | Reference (0) | Reference (1) | Reference (0) | Reference (1) | Reference (0) |
| Quartile 2 | 63,990 | 0.89 (0.82, 0.96) ^b^ | 0.56 (0.19, 0.92) ^b^ | 0.84 (0.75, 0.94) ^b^ | 0.75 (0.24, 1.25) ^b^ | 0.86 (0.73, 1.01) ^b^ | 0.59 (-0.07, 1.26) |
| Quartile 3 | 64,038 | 0.86 (0.80, 0.94) ^b^ | 0.71 (0.32, 1.09) ^b^ | 0.81 (0.72, 0.92) ^b^ | 0.93 (0.39, 1.47) ^b^ | 0.70 (0.58, 0.84) ^b^ | 1.44 (0.69, 2.19) ^b^ |
| Quartile 4 | 64,172 | 0.75 (0.68, 0.83) ^b^ | 1.34 (0.85, 1.83) ^b^ | 0.77 (0.66, 0.89) ^b^ | 1.15 (0.48, 1.82) ^b^ | 0.60 (0.46, 0.76) ^b^ | 2.04 (1.01, 3.08) ^b^ |

^a^ All models were adjusted for age, sex, education, socioeconomic status, body mass index, smoking status, alcohol drinking, physical activity, social activity, dyslipidemia, cardiovascular disease, pre-diabetes, and *apolipoprotein* E ɛ4.

^b^ *P*< 0.05.
