# Supplementary figures and images for: Estimated glucose disposal rate is associated with brain aging and dementia among diabetes-free older adults
Source: J Gerontol A Biol Sci Med Sci. 2025 Oct 31;81(1):glaf243. doi: 10.1093/gerona/glaf243 (PMC12758966; doi:10.1093/gerona/glaf243)

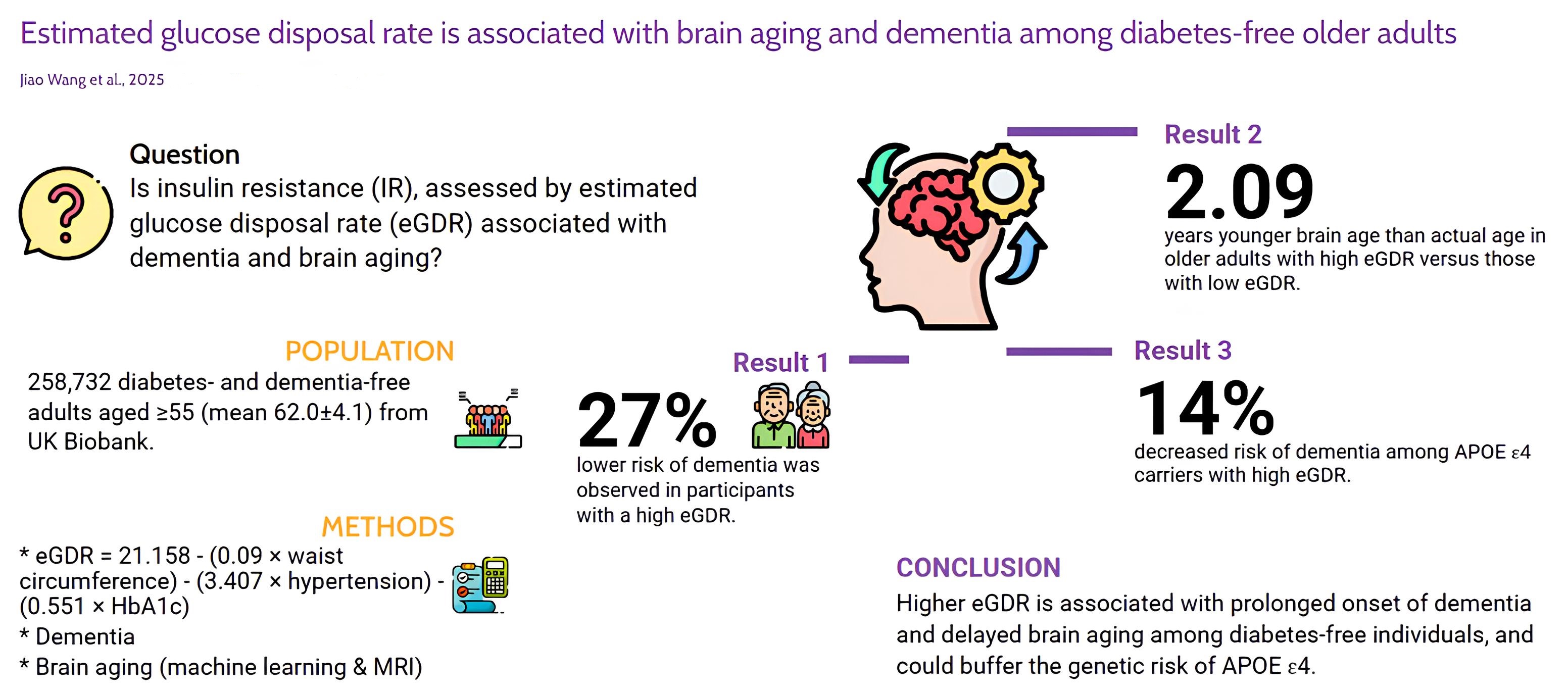

Supplement: glaf243_Supplementary_Data [file glaf243_supplementary_data.zip › graphic abstract.jpg]
